# Eight genome sequences of bacterial environmental isolates from Marr Pond, Antarctica

**DOI:** 10.1128/mra.00135-25

**Published:** 2025-05-22

**Authors:** Heidi J. Smith, Markus Dieser, Christine M. Foreman

**Affiliations:** 1 Center for Biofilm Engineering, Montana State University 33052, Bozeman, Montana, USA; 2 Department of Microbiology and Cell Biology, Montana State University 33052, Bozeman, Montana, USA; 3 Department of Chemical & Biological Engineering, Montana State University 33052, Bozeman, Montana, USA; University of Southern California, Los Angeles, California, USA

**Keywords:** Antarctica, McMurdo Dry Valleys

## Abstract

Inland meltwater ponds are common throughout the dry valley region of Antarctica, with seasonal meltwater inputs driving their biogeochemistry. Here, we report the genomic sequences of eight environmental bacterial isolates covering three major phyla from Marr Pond, Taylor Valley, Antarctica.

## ANNOUNCEMENT

Although inland meltwater ponds are common in the McMurdo Dry Valleys, their biology has received little study to date. The Nussbaum Riegel in the center of Taylor Valley, Antarctica, constricts the valley bottom into a narrow defile. The Riegel enhances the climatic delineation between the wider, lower section and the more narrow, upper section of the valley (1, 2). Above the Nussbaum Riegel (~750 m above sea level) lies a series of upland ponds.

The Marr Ponds (officially known as Kaki Ponds) consist of five ponds. The ponds are fed by meltwater from the Marr Glacier. Ponds 1–4 are hydrologically connected. The Marr Ponds have a thick ice cover with high albedo and varying moat size (3, 4). The geochemistry of these ponds is dilute, reflecting glacial meltwater inputs (4). Major ion concentration, however, can vary between ponds based on increased meltwater flushing or evaporative losses (4). Meltwater-driven changes in the size of these ponds also affect the size of the marginal wetlands and, thus, the substrate that can be exposed to chemical weathering (5). The area occupied by the Marr Ponds is among the oldest surfaces in the Taylor Valley (6). Meltwater ponds are transient and can undergo seasonal freeze-thaw cycles and thermal or chemical stratification/gradients (5). Genome features of bacteria may reflect adaptations to these strong selective pressures of the local environment.

Meltwater was collected from the Marr Ponds (77°42′S, 162°44′E) in December 2010. Bacteria were isolated aerobically on R2A agar plates at 4°C in the dark. The cetyltrimethylammonium bromide procedure was used for extracting DNA from bacterial isolates grown to the late exponential phase in R2A broth at 4°C while shaking at 150 rpm (7). Standard draft genomes were generated at JGI using the Illumina sequencing technologies (8). An input of 200 ng of genomic DNA per genome was sheared around 400 bp using the LE220-plus Focused-ultrasonicator (Covaris) and size-selected with a double SPRI method using Mag-Bind Total Pure NGS beads (Omega Bio-tek). Samples were treated with the KAPA HyperPrep kit’s (Roche) one-tube chemistry of end-repair, A-tailing, and ligation with NEXTFLEX UDI Barcodes (PerkinElmer). Illumina standard shotgun libraries were sequenced on the Illumina NovaSeq S4 platform (2 × 151 bp). Raw Illumina sequences were quality filtered using BBTools (9) per SOP 1061 and assembled with SPAdes (≥version 3.14.1; –phred-offset 33 –cov-cutoff auto -t 16 m 64 –careful -k 25,55,95) (10). Contigs with a length <1 kb (BBTools ≥version 38.86, reformat.sh: minlength = 1000 ow = t) were discarded. CRISPR elements were identified using the program CRT version 1.8.2 (11). All genomes were annotated in the Integrated Microbial Genomes (IMG) database (≥IMG Annotation Pipeline version 5.0.19) (12). Genome completeness was estimated using checkM version 1.2.2 (13). The genome-sequence-acquired 16S ribosomal RNA gene was queried in command line against the SILVA database 138.1 with blastn, from BLAST+ 2.13.0 for taxonomic classification (14). Sequence details are given in Table 1.

**TABLE 1:** Summary of genome characteristics^*a*^

Organism (% match to genus, accession #) accession number	# reads	Coverage (×)	Completeness (%)	Contamination (%)	# contigs	*L*_50_/*N*_50_ (bp)	Size (bp)	GC (%)	# genes	# C RISPR
*Arthrobacter* sp. MP_2.3 (99.4; NR_178602.1) JBJXHZ000000000 SRR30853865	44,764,632	380.4	99.7	0.0	88	11/86,476	4,038,123	65.98	3,918	1
*Cryobacterium* sp. MP_3.1 (99.3; NR_108605.1) JAXCHV000000000 SRR25764249	10,570,838	407.5	99.5	1.52	82	11/113,724	3,899,650	67.91	3,760	1
*Chryseobacterium* sp. MP_3.2 (99.5; KY476414.1) JAXCHW000000000 SRR25764257	10,587,412	517.7	100	0.0	20	4/214,192	2,896,799	35.72	2,872	1
*Massilia* sp. MP_M2 (99.6; NR_042502.1) JBCHJF000000000 SRR25764246	10,124,380	291.7	100	0.2	27	4/481,589	5,390,661	63.50	4,868	–
*Cryobacterium* sp. MP_M3 (98.9; NR_043892.1) JADOVI000000000 SRR13164991	9,144,084	355.8	99.5	1.01	56	6/231,442	3,940,246	68.44	3,724	1
*Arthrobacter* sp. MP_M4 (99.3; NR_178602.1) JAXCHX000000000 SRR25764245	7,644,976	285.4	99.7	0.0	73	10/111,605	4,093,838	65.87	3,903	–
*Cryobacterium* sp. MP_M5 (90.0; NR_043892.1) JAXCHY000000000 SRR25764250	14,177,762	395.8	99.5	1.01	63	7/175,936	3,941,243	68.45	3,732	1
*Arthrobacter* sp. MP_M7 (99.5; NR_178602.1) JAXCHZ000000000 SRR25764255	11,663,040	373.9	99.7	0.0	76	11/116,442	4,096,488	65.87	3,895	–

^*a*^ –, none.

## Data Availability

The work conducted by the U.S. Department of Energy Joint Genome Institute, a DOE Office of Science User Facility, is supported under Contract No. DE-AC02-05CH11231. These data were generated for JGI Proposal #505037. The genome sequences have been submitted to GenBank and are available through the accession numbers listed in Table 1.

## References

[1] Fountain AG, Lyons WB, Burkins MB, Dana GL, Doran PT, Lewis KJ, McKnight DM, Moorhead DL, Parsons AN, Priscu JC, Wall DH, Wharton RA, Virginia RA. 1999. Physical controls on the Taylor Valley ccosystem, Antarctica. Bioscience 49:961. doi:10.2307/1313730

[2] Fountain AG, Nylen TH, Monaghan A, Basagic HJ, Bromwich D. 2010. Snow in the McMurdo dry valleys, Antarctica. Intl Journal of Climatology 30:633–642. doi:10.1002/joc.1933

[3] Moorhead DL, Barrett JE, Virginia RA, Wall DH, Porazinska D. 2003. Organic matter and soil biota of upland wetlands in Taylor Valley, Antarctica. Polar Biol 26:567–576. doi:10.1007/s00300-003-0524-x

[4] Lyons WB, Welch KA, Gardner CB, Jaros C, Moorhead DL, Knoepfle JL, Doran PT. 2012. The geochemistry of upland ponds, Taylor Valley, Antarctica. Antarct Sci 24:3–14. doi:10.1017/S0954102011000617

[5] Healy M, Webster-Brown JG, Brown KL, Lane V. 2006. Chemistry and stratification of Antarctic meltwater ponds II: Inland ponds in the McMurdo Dry Valleys, Victoria Land. Antartic science 18:525–533. doi:10.1017/S0954102006000575

[6] Marchant DR, Denton GH. 1996. Miocene and Pliocene paleoclimate of the Dry Valleys region, Southern Victoria land: a geomorphological approach. Mar Micropaleontol 27:253–271. doi:10.1016/0377-8398(95)00065-8

[7] William S, Feil H, Copeland A. 2012. Bacterial genomic DNA isolation using CTAB. Sigma 50:6876.

[8] Bennett S. 2004. Solexa Ltd. Pharmacogenomics 5:433–438. doi:10.1517/14622416.5.4.433 15165179

[9] Bushnell B. 2022 BBTools Software package (version 38.95. https://bbtools.jgi.doe.gov.

[10] Bankevich A, Nurk S, Antipov D, Gurevich AA, Dvorkin M, Kulikov AS, Lesin VM, Nikolenko SI, Pham S, Prjibelski AD, Pyshkin AV, Sirotkin AV, Vyahhi N, Tesler G, Alekseyev MA, Pevzner PA. 2012. SPAdes: a new genome assembly algorithm and its applications to single-cell sequencing. J Comput Biol 19:455–477. doi:10.1089/cmb.2012.0021 22506599 PMC3342519

[11] Bland C, Ramsey TL, Sabree F, Lowe M, Brown K, Kyrpides NC, Hugenholtz P. 2007. CRISPR recognition tool (CRT): a tool for automatic detection of clustered regularly interspaced palindromic repeats. BMC Bioinformatics 8:1–8. doi:10.1186/1471-2105-8-209 17577412 PMC1924867

[12] Chen I-MA, Chu K, Palaniappan K, Pillay M, Ratner A, Huang J, Huntemann M, Varghese N, White JR, Seshadri R, Smirnova T, Kirton E, Jungbluth SP, Woyke T, Eloe-Fadrosh EA, Ivanova NN, Kyrpides NC. 2019. IMG/M v.5.0: an integrated data management and comparative analysis system for microbial genomes and microbiomes. Nucleic Acids Res 47:D666–D677. doi:10.1093/nar/gky901 30289528 PMC6323987

[13] Parks DH, Imelfort M, Skennerton CT, Hugenholtz P, Tyson GW. 2015. CheckM: assessing the quality of microbial genomes recovered from isolates, single cells, and metagenomes. Genome Res 25:1043–1055. doi:10.1101/gr.186072.114 25977477 PMC4484387

[14] Quast C, Pruesse E, Yilmaz P, Gerken J, Schweer T, Yarza P, Peplies J, Glöckner FO. 2013. The SILVA ribosomal RNA gene database project: improved data processing and web-based tools. Nucleic Acids Res 41:D590–6. doi:10.1093/nar/gks1219 23193283 PMC3531112

